# Digestibility and IgE-Binding of Glycosylated Codfish Parvalbumin

**DOI:** 10.1155/2013/756789

**Published:** 2013-06-26

**Authors:** Harmen H. J. de Jongh, Carlos López Robles, Eefjan Timmerman, Julie A. Nordlee, Poi-Wah Lee, Joseph L. Baumert, Robert G. Hamilton, Steve L. Taylor, Stef J. Koppelman

**Affiliations:** ^1^TI Food and Nutrition, P.O. Box 557, 6700 AN Wageningen, The Netherlands; ^2^Food Physics Group, Department for Agrotechnology and Food Science, Wageningen University, P.O. Box 557, 6700 AN Wageningen, The Netherlands; ^3^Food Allergy Research and Resource Program, Food Science and Technology, University of Nebraska-Lincoln, 257 Food Industry Complex, Lincoln, NE 68583-0919, USA; ^4^Department of Medicine, Division of Allergy & Clinical Immunology, Johns Hopkins Asthma & Allergy Center, 5501 Hopkins Bayview Circle, Room 1A.20, Baltimore, MD 21224-6801, USA

## Abstract

Food-processing conditions may alter the allergenicity of food proteins by different means. In this study, the effect of the glycosylation as a result of thermal treatment on the digestibility and IgE-binding of codfish parvalbumin is investigated. Native and glycosylated parvalbumins were digested with pepsin at various conditions relevant for the gastrointestinal tract. Intact proteins and peptides were analysed for apparent molecular weight and IgE-binding. Glycosylation did not substantially affect the digestion. Although the peptides resulting from digestion were relatively large (3 and 4 kDa), the IgE-binding was strongly diminished. However, the glycosylated parvalbumin had a strong propensity to form dimers and tetramers, and these multimers bound IgE intensely, suggesting stronger IgE-binding than monomeric parvalbumin. We conclude that glycosylation of codfish parvalbumin does not affect the digestibility of parvalbumin and that the peptides resulting from this digestion show low IgE-binding, regardless of glycosylation. Glycosylation of parvalbumin leads to the formation of higher order structures that are more potent IgE binders than native, monomeric parvalbumin. Therefore, food-processing conditions applied to fish allergen can potentially lead to increased allergenicity, even while the protein's digestibility is not affected by such processing.

## 1. Introduction

Fish allergies are common in North America, Europe, and Asia and can potentially be fatal [1]. Several population-based studies on the prevalence of fish allergy have been performed in mainly western countries indicating a prevalence of about 0.5% [2, 3]. Parvalbumin is considered a panallergen for fish allergic patients [4, 5] as it shares significant biochemical and immunochemical similarity across fish species consumed in western countries.

Parvalbumins are proteins conserved in lower vertebrates that occur in relatively high amounts in white muscle. Parvalbumins are also found in the fast twitch skeletal muscles of higher vertebrates, as well as in a variety of nonmuscle tissues, including testis, endocrine glands, skin, and specific neurons [6]. The main function of parvalbumin in fish is in the muscle contraction/relaxation cycle, calcium buffering, and signal transduction. Parvalbumins are typically 10–12 kDa in size and acidic (pI = 4.0–5.2). They are structurally characterized by the presence of three typical helix-loop-helix domains (EF hand domain), two of which are able to bind divalent cations, like Ca^2+^ [7]. Parvalbumin is a relatively abundant protein in muscle tissue, and for cod it is estimated that 0.15 to 0.625% of the fish muscle tissue (wet weight) is parvalbumin [8, 9].

Food allergens in general share the characteristic that they are resistant to digestion. Poor digestibility is associated with a high sensitizing potential, as limited digestion in the gastrointestinal tract results in rather large, potentially immunogenic peptides that are then exposed to the gut's immune system [10]. Once sensitized, a food allergic individual may experience an allergic reaction upon consumption of the offending food. Binding of immunoglobulin E (IgE) antibodies is essential to elicit an allergic reaction. IgE-binding can occur in the oral cavity causing localized allergic reactions that are typically mild, or they can occur after uptake by the gastrointestinal tract, causing systemic allergic reactions involving multiple organs that can be more severe. Uptake and systemic reactions are more likely for allergens that are resistant to digestion in the gastrointestinal tract. Codfish allergens have a grossly reduced ability to trigger an intestinal allergic reaction when they are digested [11], and incomplete gastric digestion of cod allergens represents a risk factor for allergen-induced anaphylaxis [12]. The ingestion of antacids, an increasingly common practice in the western world, can increase the stomach pH leading to enhanced sensitizing potential *in vivo* [13]. 

Thermal processing of foods may induce protein denaturation, potentially leading to altered physicochemical characteristics, digestibility, and allergenicity. Fish can be consumed as fresh, raw meat (sushi), as a heated product (canned to extend shelf life), or as fresh fish cooked at home or by a caterer. Moreover, fish protein is widely applied as an ingredient in complex foods to provide nutritional value or texture to the product. This is of particular importance in the new economies in Asia. In many cases, fish or fish products are marinated to improve their taste and texture. Marinating can be done with salt, acid, sugar, or a combination of these ingredients potentially leading to Maillard reactions when the fish is subjected to heat processing. The Maillard browning reaction occurs during heat processing when lysine (Lys)-residues in tissue protein chemically react with sugars that are present especially after marination.

The current study aims to investigate the effect of glycosylation on the digestibility and IgE-binding of codfish parvalbumin. We tested the digestibility of parvalbumin at different pHs., and we characterized the resulting peptides biochemically and immunochemically.

## 2. Materials and Methods

### 2.1. Parvalbumin

Parvalbumin was purified from Atlantic cod (*Gadus morhua*), using a protocol that avoids heat treatment, as was published for carp parvalbumin [14]. Briefly, codfillets were extracted in a 38 mM TRIS buffer (pH = 8) and diafiltered to obtain a 3- to 30-kDa fraction. This fraction was further purified to homogeneity by anion exchange and size exclusion chromatography resulting in an estimated purity of parvalbumin of 95–98% (results not shown). It was stored frozen until further use.

### 2.2. Glycosylation of Parvalbumin and Biochemical Characterization

Parvalbumin was glycosylated as previously described by De Jongh and coworkers [15] with minor modifications in the final dialysis step: parvalbumin was dialyzed in centrifuge tubes using a 5000 Da molecular weight cut-off filter (Vivaspin 15R, Sartorius, Germany). Different batches were prepared by incubating at 60°C with glucose (molar ratio of primary amino groups : glucose = 1 : 5) for 5, 12, 24, and 48 hours, respectively. Control batches were prepared by incubating under the same conditions in absence of glucose, or in the presence of sucrose, a nonreducing sugar, instead of glucose. Materials were stored at −20°C till further use. The degree of glycosylation was determined by quantifying free amino groups applying ortho-phthaldialdehyde (OPA) as described earlier [16]. Matrix Assisted Laser Desorption/Ionization-Time of Flight Mass Spectrometry (MALDI-TOF MS) was used to assess the mass of proteins and peptides, using settings described earlier [16]. Sequences of cod parvalbumin as published in UniProtKB (Q90YL0 and Q90YK9) were used to match masses and sequences. MALDI-TOF MS, isoelectric focusing (IEF), far UV CD spectroscopy, and intrinsic fluorescence spectroscopy were performed using protocols published earlier [15, 17]. For CD spectroscopy at different pHs, the following solutions were used. For pH 1.2, a 25 mM KCl/0.063 M HCl; for pH 2, a 5 mM phosphate buffer; for pH 3, a 5 mM citrate buffer; for pH 4, a 5 mM acetate buffer; and for pH 5.5, a 5 mM succinate buffer. Buffers were used at such low concentration to avoid distortion of the spectra at lower wavelengths, especially at low pH. Measurements were performed at 37°C (typically ±1°C) using controlled water baths or Peltier elements. 

### 2.3. **In Vitro ** Digestion

The digestion assay was performed in simulated gastric fluid (SGF), with the conditions established by Thomas and coworkers [18]. The assay was slightly modified for this experiment by using a larger reaction volume (2 mL total volume), which accommodates multiple sampling over time. 0.1 mL of a 5 mg/mL parvalbumin (native or glycosylated) solution was mixed with 1.9 mL of SGF containing 35 mM of NaCl and 0.064 N HCl for the sample at pH 1.2. The amount of HCl needed to reach the different pHs (1.2, 2, 3, 4, and 5.5) varied. At each pH, three concentrations of pepsin per microgram of parvalbumin were tested: 1 U, 0.1 U, or 0.01 U (Sigma, 3802 U/mg). Samples (200 *μ*L) were taken at the following time points: *t* = 0, 0.25, 0.5, 1, 2, 4, 8, 15, 30, and 60 minutes, and the reaction was stopped as described by neutralizing and mixing with SDS-PAGE sample buffer [18]. Samples were stored at −20°C until further analyzed. Digestions were performed 2 times independently; figures show a typical example.

### 2.4. Patient Serum

Deidentified and discarded sera from 21 individuals with a positive history of immediate-type hypersensitivity reactions to fish were obtained from the Johns Hopkins University Dermatology, Allergy and Clinical Immunology Reference Laboratory, Baltimore, Maryland, USA. At the time of collection, all individuals provided general consent for use of their serum in research studies related to food allergy. All sera (0.5 to 3 mL per individual) were initially analyzed for cod-specific IgE by Immuno-CAP (Thermofisher Scientific/Phadia, Uppsala, Sweden). Sera (*n* = 16) with cod-specific IgE >5 kU/L [range: 5.7 to >100 kU/L (mean: 25.3 kU/L)] and with at least 1 mL of volume were pooled equivolumetric quantities. This pool was used in the immunoblotting studies.

### 2.5. SDS-PAGE and IgE-Immunoblot Blot

SDS-PAGE was performed as described previously for the characterization of peanut allergen digests [19], in this case, 1.5 *μ*g per lane was loaded. Densitometric analyses were conducted to quantify the band intensities using a densitometer GS-710 from Biorad (Veenendaal, the Netherlands, model GS-710), and the data were corrected for blank gelsections on the same gel. Gates were set such that the target protein bands (at approximately 3, 4, and 10 kDa) were not overlapping. Values of intensity of each band were compared with the summed density in the whole lane and reported as percentages (relative quantity). The sum of the shown intensities of the 3, 4, and 10 kDa bands is therefore not necessarily 100%. For IgE-immunoblotting, gels were electroblotted onto PVDF membranes. Protein transfer was confirmed using Ponceau Red (Sigma Chemical, St. Louis, MO, USA). Membranes were blocked for 2 h using 5% (W/V) nonfat dry milk in wash buffer consisting of 0.01 M sodium phosphate buffered saline, pH 7.4 containing 0.05% Tween. The serum pool was diluted 1 : 10 in wash buffer +2.5% (W/V) nonfat dry milk and preincubated at room temperature for 1 hr before adding to the washed, blocked membranes for overnight incubation. After washing 4 times with wash buffer, the membranes were incubated with mouse anti-human IgE conjugated with horseradish peroxidase (Southern Biotech, Birmingham, AL USA) diluted 1 : 1000 (v/v) in wash buffer +2.5% (W/V) nonfat dry milk for 1 hr at room temperature. The blots were washed (4X with wash buffer), and substrate solution was added (SuperSignal West Dura Extended Duration Substrate Kit, Thermo Pierce Chemical, Rockford, IL, USA). Emitted light was detected using a Kodak Gel Logic 440 image station, and the resulting images were stored digitally.

## 3. Results and Discussion

### 3.1. Degree of Modification of Glycosylated Parvalbumin

**Figure 1:**
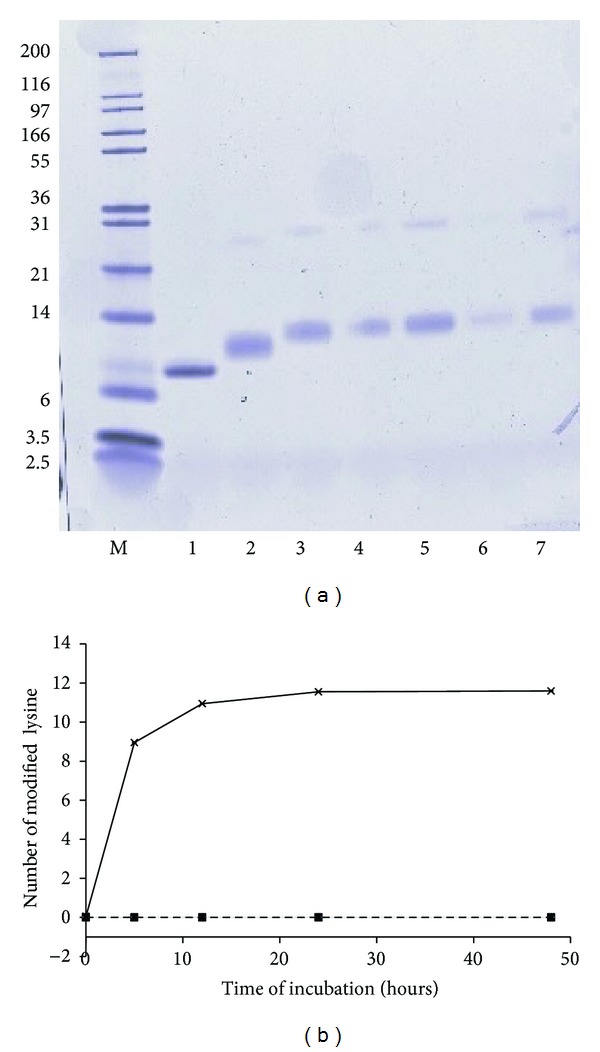
Modification of parvalbumin. (a) *SDS-PAGE analysis of parvalbumin. *M: marker, indicated in left margin in kDa. Lane 1: native; Lane 2: 5-hour glycosylated; Lane 3: 12-hour glycosylated; Lane 4: 24-hour glycosylated; lane 5: as lane 4 with double amount of protein loaded; Lane 6: 48-hour glycosyated; lane 7: as lane 6 with double amount of protein loaded. (b) *Degree of modification determined via free lysine analysis. *Number of modified lysine residues per mole of parvalbumin after reaction with glucose (black line), sucrose (grey line), and nontreated (dashed line). Lines of nontreated parvalbumin and sucrose-treated parvalbumin lines are at zero and overlaid. Standard deviations are <0.02 and not plotted.

Figure 1(a) shows the molecular weight (MW) of the different samples of parvalbumin (native and glycosylated) under reducing conditions. Upon glycosylation, a shift to higher apparent MW is observed reaching a plateau upon prolonged incubation of 24 hrs. Also a band at an apparent MW of around 20–25 kDa is observed upon glycosylation, most likely representing dimers of parvalbumin. Dimers and higher order multimers have been described in fish extracts and may be induced by denaturing conditions [20, 21]. In Figure 1(b), the degree of glycosylation as a function of incubation time is shown. This was determined by measuring the number of residual free amino groups. After 5 hrs, 9 of the available 12 Lys residues appear to be glycosylated, and this number increases to 11 by 12 hours. This is consistent with the increase in MW that is observed in Figure 1(a). Control incubations in the absence of glucose or in the presence of the nonreducing sugar sucrose do not give rise to glycosylation (Figure 1(b)) or an increase in the apparent MW (not shown). Summarizing, a 12-hour incubation under the chosen conditions leads to a close to complete glycosylation of parvalbumin, while a 5-hour treatment leads to a partially glucosylated parvalbumin. 

Given the high degree of Lys modifications in the Maillard-treated parvalbumin, it was speculated that its isoelectric point (IEP) would be substantially lowered. Isoelectric focusing showed, however, only a minor shift in IEP of 0.1 pH units towards being more acidic as a result of the glycosylation (data not shown). Similar observations were made for beta-lactoglobulin [16] and are attributed to the shielding effect of the protein surface charges by the sugar moieties, thereby minimizing the effect of the modification on the apparent IEP.

**Figure 2:**
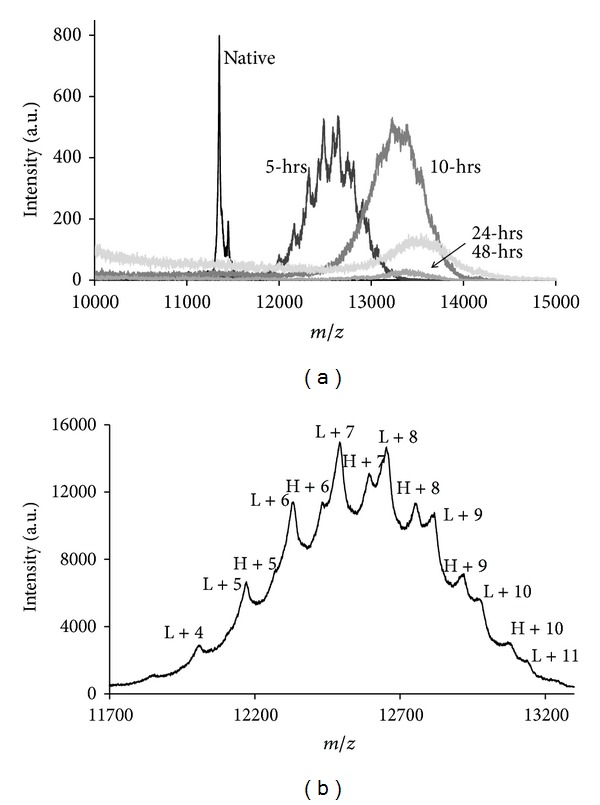
MALDI-ToF MS of different forms of Parvalbumin. (a) Spectra of different glycosylated samples. (b) Spectrum of 5-hour treated parvalbumin, indicated are the peaks corresponding to the light isoform (L) and heavy isoform (H) with the corresponding numbers of glucose added.

Figure 2(a) shows the mass range of 10,000 to 15,000 Da for the native and the glycosylated forms of parvalbumin as determined with MALDI-TOF mass spectroscopy. There are several isoforms for cod parvalbumin reported in protein sequence databases (UniProt entries: Q90YK9, Q90YL0, A5I874, A5I873, and P02622). Considering that mature cod parvalbumin is missing the N-terminal Met residue (−132 Da) and that the N-terminus is acetylated (+43 Da, UniProt), the theoretical masses can be determined. The experimental mass of the main peak was 11,360, which corresponds to Q90YL0 rather than to A5I873, with mass differences of 5 and 19 Da, respectively. The second peak at 11,459 Da corresponds equally well to Q90YK9 and to A5I874, with mass differences of 3 and 4 Da, respectively. Unambiguous identification of specific isoforms is therefore not possible. 

Addition of a single glucose to a Lys residue results in a mass addition of 162 Da. The average degrees of modification of 9 and 11 reached at 5 and 12 hours of treatment, respectively correspond with an average mass increase of 1,458 Da for the 5-hour-treated sample, and 1,782 Da for the 12-hour-treated sample. Mass spectroscopy on the 12-hour-treated sample show mass increase in line with this calculation, while for the 5-hour-treated sample, the average mass shift seems less than the expected (Figure 2(a)). Overall, the average mass increases with the incubation time, and the peaks become broader, less resolved, and lower in intensity (Figure 2(a)). The less resolving peaks can be explained by continuation of the Maillard reaction. After the initial addition of glucose, the reaction product may undergo Amadori rearrangement leading to complex and less defined structures (advanced glycosylated end products; AGEs) as was earlier described in detail for another food protein [22]. Such molecules may be less soluble and more difficult to ionize, which may explain the lower overall intensities of the MALDI-TOF MS spectra produced by the 24- and 48-hour-treated samples.  Figure 2(b) shows the spectrum of the 5-hour-treated parvalbumin, zoomed-in to the range of 11,500 to 13,500 Da. Using the experimental masses of the two isoforms of native parvalbumin (11,360 and 11,459 Da), all main peaks could be assigned to integer numbers of Lys residues modified. Gaussian distributions for degree of modification have also been reported for other proteins treated by the glycosylation [17]. One should take into account that MALDI-TOF is not a quantitative method. Thus, no relative percentages could be attributed to the different degrees of modification. Some peaks of the spectrum of the 12-hour-treated parvalbumin could be assigned in a similar manner; however, the low resolution of the spectrum due to AGEs hampers such analysis. 

### 3.2. Structural Properties of Native and Glucosylated Parvalbumin at Ambient and Low pH

**Figure 3:**
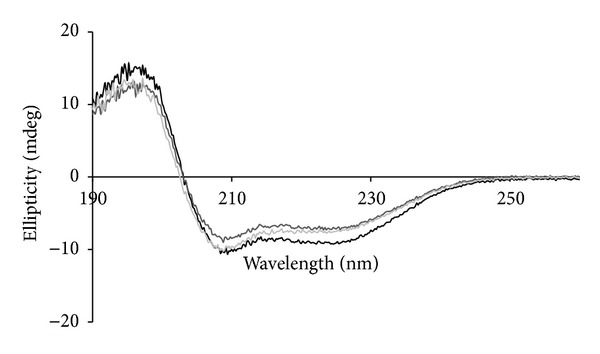
Secondary structure content of different forms of parvalbumin. Far UV circular dichroism spectra of native (black line), 5 hour glycosylated (dark grey line), and 12-hour glycosylated parvalbumin (grey line) at neutral pH.

The far UV CD spectrum of native parvalbumin (Figure 3) resembles that of cod parvalbumin as previously published [23] with minima at 208 and 222 nm and a zero-crossing around 204 nm. Such a shape is typical for a high content of alpha-helix and some beta structures because of the high zero-crossing and the substantially deeper minimum at 208 nm than that at 222 nm. Most prominent is the steep increase to the lower wavelengths (from 208 nm downwards), indicative of a high content of alpha-helices. Both the 5-hour- and 12-hour-treated parvalbumin samples display analogous spectral characteristics (Figure 3), indicating a secondary structure content comparable to that of native parvalbumin. The difference in intensity is due to a slight difference in protein concentration. 

Because we intend to investigate the digestibility at low pH, we investigated whether decreasing the pH alters the overall secondary structure of the different forms of parvalbumin. Far UV-CD spectra were recorded from 190 to 260 nm at various pHs. At pH 1.2, the spectra could not be recorded properly at the low wavelengths because of the high absorbance by chloride required to reach this pH. Also at pH 2, a relative high noise was observed in the spectra below 195 nm (not shown). The spectra of all samples at the various pHs were comparable, though some differences were observed at the extreme low pH values. To facilitate comparison of spectra, the wavelength at which the signal crosses 0 ellipticity (zero-crossing) is investigated. Zero-crossing is considered a relevant marker for conformational changes of proteins rich in alpha-helix and beta structures. A shift of zero-crossing to lower wavelength indicates loss of alpha-helix content [24]. Zero-crossing values for the three parvalbumin samples at different pHs are summarized in Table 1. The shift of zero-crossing to lower wavelengths for native parvalbumin is associated with unfolding of the protein, as was earlier demonstrated for the heat-induced denaturation of carp parvalbumin [23]. This shift is also observed for the glycosylated parvalbumin, although to a lesser extent. At pH 4, the native parvalbumin had a distorted far UV CD spectrum with a zero-crossing at 207 nm and absence of the minimum at 208 nm. This is probably due to isoelectric destabilization at this pH close to the IEP, which results in an enhanced beta-structure formation, possibly related to (reversible) protein self-association. This was not observed for the glycosylated parvalbumin samples. Apparently, the linkage of hydrophilic sugar moieties to parvalbumin helped to stabilize at pHs close to the IEP, as was earlier described for beta-lactoglobuin from bovine milk [17].

Information on tertiary protein folding was obtained by intrinsic fluorescence spectroscopy. At neutral pH a single peak around 330 nm was observed, representing fluorescence of tryptophans that are readily shielded from the aqueous solvent, for native and glycosylated parvalbumins (not shown). At low pH (2.0 and 1.2), the emission maximum for native parvalbumin is found at 328 nm, whereas for glycosylated parvalbumin, a distinct red-shifted emission maximum (340 nm) was observed, indicating a slightly more water-exposed environment for tryptophan. A fully exposed tryptophan would exhibit a maximum around 355 nm. A moderate red-shift could thus reflect a slightly destabilized tertiary fold, without necessarily a loss of secondary structure. 

Summarizing, analysis indicates that native parvalbumin is structurally sensitive to low pH conditions and around its IEP, while glycosylation provides some protection against such sensitivity.

### 3.3. Digestibility of Native and Glycosylated Parvalbumin upon Digestion at Different pHs

**Figure 4:**
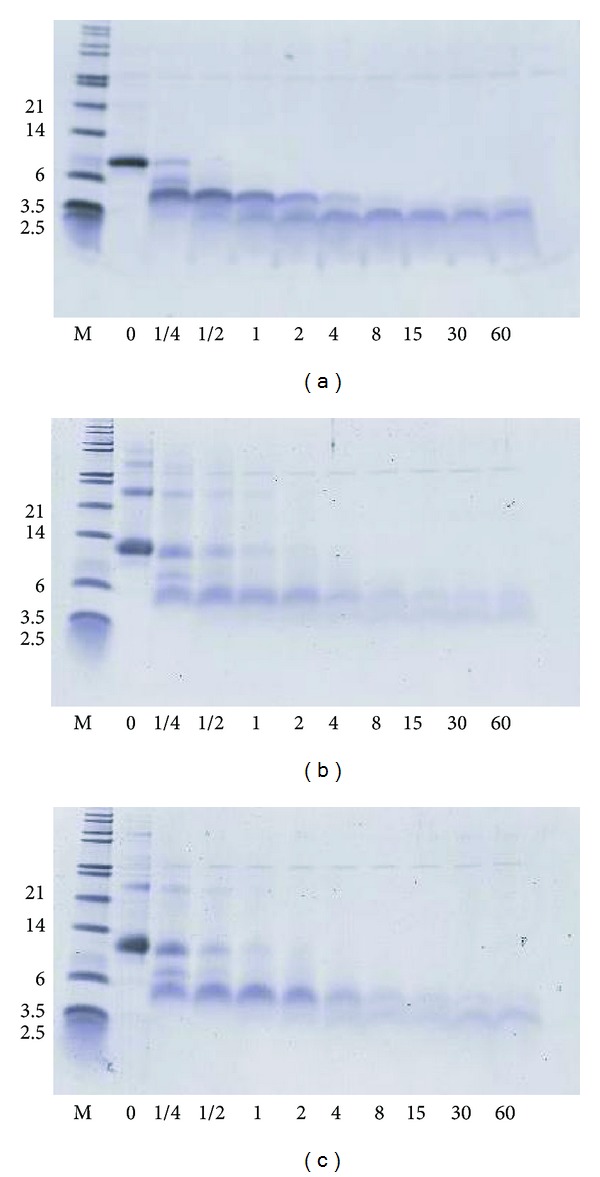
Time course of digestion of different forms of parvalbumin at pH. (a) Native; (b) 5 hour glycosylated; (c) 12 hour glycosylated. Incubation times are shown at the bottom of the gels (minutes). MW markers are indicated in left margin (kDa).

Figure 4 shows the protein profile of the digestions of native parvalbumin, 5 hour Maillard-treated parvalbumin and 12 hour Maillard-treated parvalbumin at pH 2 using 0.1 Units of pepsin per microgram of parvalbumin. The faint band at approximately 40 kDa is pepsin. For all three samples, substantial proteolysis was already observed after 1 minute; however, the peptides that result from this proteolysis remain for prolonged incubation times. Digestibility of food proteins has been studied intensively. An initial *in vitro* study using plant proteins showed that allergenic food proteins from peanut and soy were stable toward digestion [25]. In contrast, non-allergenic ribulose bis-phosphate carboxylase/oxygenase from spinach leaves (Rubisco) was rapidly digested [25] which was later confirmed in a multi-laboratory investigation [18]. We used a 100-fold lower pepsin/substrate ratio because preliminary data showed that the higher ratio resulted in a rate of proteolysis that was too fast. Applying the ratio used earlier, parvalbumin displayed breakdown kinetics between that of peanut/soy proteins and Rubisco [18]. One study on the digestion of peanut proteins applied the same ratio of pepsin/substrate as we used here [19]. Peanut allergens Ara h2 and Ara h6 were stable during the entire incubation time of 90 minutes, while the other peanut allergens Ara h1 and Ara h3 were degraded at about the same rate as we observe here for parvalbumin. Parvalbumin can thus be classified as a protein that is moderately stable in relation to digestion. For glycosylated parvalbumin, it was observed that the bands of the glycosylated samples are more diffuse than those of native parvalbumin. Also multimers can be observed in Maillard-treated samples (see also Figure 1). With regard to the kinetics of digestion, there are only minor differences. The intact band of native parvalbumin disappears more quickly, and the resulting peptides of native parvalbumin have a higher resistance to further degradation. There are no substantial differences with regard to peptide diversity based between native and glycosylated parvalbumins based on the SDS-PAGE analysis. One should keep in mind that SDS-PAGE analysis has limited resolution, in particular in the low molecular weight region, making it difficult to draw firm conclusion on peptides masses.

**Figure 5:**
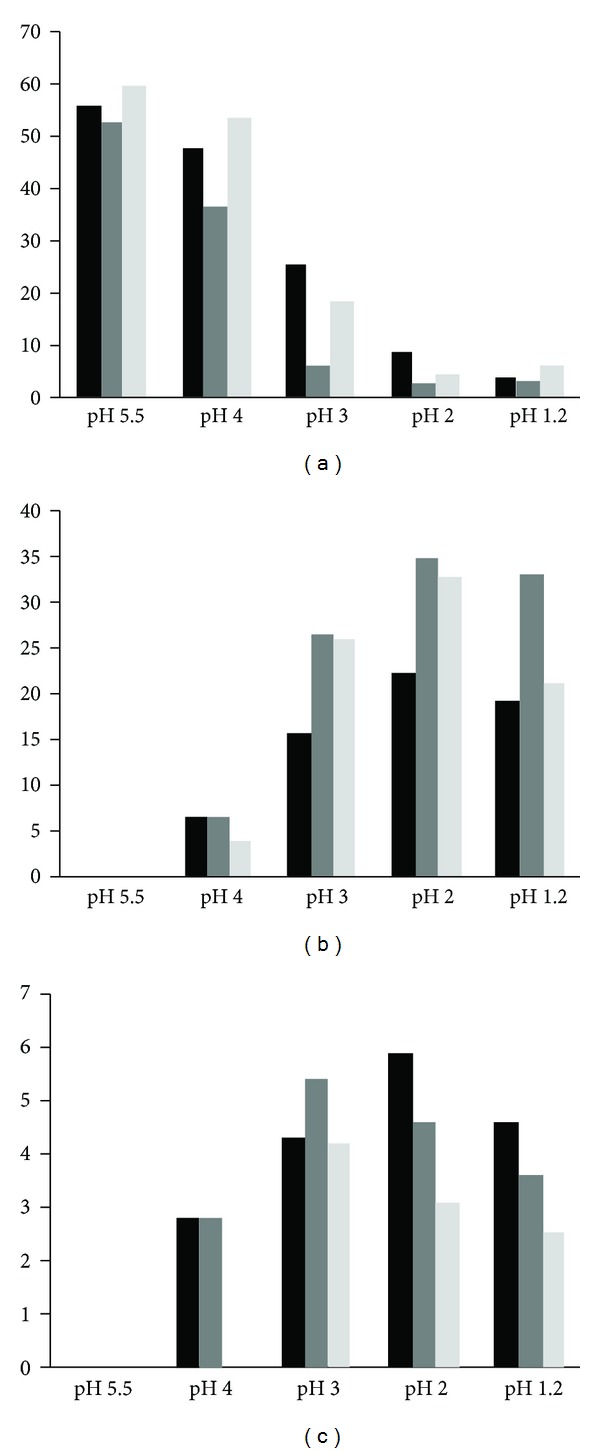
pH dependency of digestion of different forms of parvalbumin. The relative amount of intact parvalbumin (a), the 4 kDa peptide (b), and the 3 kDa peptide (c) were determined at one minute of digestion at different pHs using densitometry. Black bars: native parvalbumin; Dark grey bars: 5-hour glycosylated parvalbumin; light grey bars: 12-hour glycosylated parvalbumin.

The use of acid-suppression medication has become more common in western countries in the last decades. Symptoms of gastritis, ulcer, erosions, and reflux syndromes can be treated with medication that are available over-the-counter, that is, without prescription by a physician. Such drugs can increase the pH of the stomach up to pH 5, thereby limiting the individual's digestive capacity. It has been hypothesized that the more common use of acid-suppressing drugs is associated with the increasing prevalence of food allergies [26], but no prospective data in humans are available to support this theory. Preliminary reports on the oral sensitization of mice treated with acid-suppressive drugs tend to support the suggestion that an elevated stomach pH can increase the risk of sensitization to food proteins [27]. It has been shown by Untersmayr et al. that a codfish extract, containing parvalbumin as well as other codfish muscle proteins, was digested well at pH <2.75 but at pHs above this value, there was virtually no digestion [11]. When carefully reviewed, that data of Untermayr et al. show that parvalbumin is present in digestion mixes prepared at all pHs. The parvalbumin band is less abundant in sample digested at low pH (1.25, 2, and 2.5) but not absent [11]. Interpretation of the gels by Untersmayr is hampered by the presence of other codfish tissue proteins resulting in proteins and peptides that comigrate with parvalbumin. Together, this indicates that, while other codfish muscle proteins digest well at low pH, parvalbumin (or large fragments thereof) remains present after digestion. Probably the pepsin concentration was not sufficient in those studies to see complete disappearance of the parvalbumin band upon digestion. We wanted to investigate the digestibility of native and glycosylated parvalbumins at pHs ranging from 1.2 up to neutral pH, covering the range of pH that can occur in humans. The digestion experiment shown in Figure 4 was repeated at pHs 1.2, 2, 3, 4, and at neutral pH (as a control). Band intensities of the intact bands and resulting peptides after one minute of digestion were quantified by densitometry and are summarized in Figure 5. For further investigation, we selected two main peptides that resulted from the digestion (at approximately 4 kDa and 3 kDa). At pHs 1.2 and 2, the parvalbumin band for all three preparations disappears almost completely in one minute, and the peptides of approximately 4 kDa and 3 kDa appear. At pH 1.2, which is our optimal experimental condition, less of the resulting peptides are observed, probably because the digestion continues breaking down the resulting peptides within one minute of incubation. Indeed at pH 2, the presence of the 3 and 4 kDa bands is more important than at pH 1.2 (Figure 5). With increasing pH, the disappearance of the intact band becomes less pronounced, and consequently the appearance of the 3 and 4 kDa peptides (for both native and glycosylated parvalbumins) becomes less evident. There are some differences between the native and glycosylated samples, but these are minor. Taken together, we see strong pH dependency for the digestion for both native and glycosylated parvalbumins, indicating that pepsin digestion up to pH 3 is efficient but not at higher pH values.

MALDI-ToF MS was applied to further characterize the peptides. However, due to the complexity of the degradation pattern of the multiple isoforms together with variable glycosylations, made an unambiguous assignment was not feasible. This latter was, for example, illustrated in the mass spectrum of the digested sample of the 5-hour glycosylated parvalbumin where a series of peaks separated 162 Da from each other could be observed (not shown), which indicates the same peptide with different degrees of modification. 

### 3.4. IgE-Binding Properties of Native and Glycosylated Parvalbumins and Their Pepsin-Resistant Peptides

**Figure 6:**
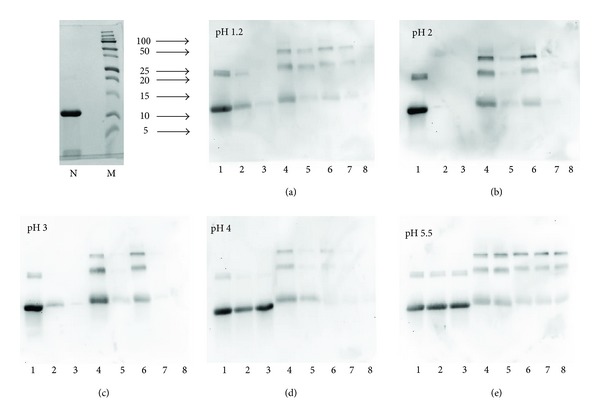
IgE reactivity of different forms of parvalbumin digested at various pHs. Panels show different pH applied during digestion, indicated in upper right corner of each panel. Lane 1: Native undigested; Lane 2: native digested for 1 minute; Lane 3: Native digested for 8 minutes; Lane 4: 5 hour glycosylated undigested; Lane 5: 5-hour glycosylated digested for 1 minute; Lane 6: 12-hour glycosylated undigested; Lane 7: 12-glycosylated digested for 1 minute; Lane 8: 12-hour glycosylated PV digested for 8 minutes. Inset upper left corner: Coomassie-stained SDS-PAGE of native parvalbumin (N) and marker proteins (M) used for immune-blot shown in (a)–(e). Molecular weights are indicated in kDa.

The rather stable peptides that are obtained after digestion are of sufficient size to comprise IgE epitopes, because the molecular weights of 3 and 4 kDa (Figure 4) correspond to polypeptides of about 25 to 40 amino acids. For a hypoallergenic food product such as hydrolyzed milk-based infant formulae, the targeted peptide weight is 1,000 and 3,000 Da for “partially” hydrolyzed formulae and below 1,000 Da for “extensively” hydrolyzed formulae [28]. The peptides found in this study at 3 to 4 kDa may therefore be potentially allergenic. We have evaluated the IgE-binding properties of the digestion products by means of IgE immunoblotting, using serum from 16 patients with fish allergy as source of IgE.  Figure 6 shows the IgE-binding to the digested native parvalbumin and, 5-hour- and 12-hour glycosylated parvalbumin. While on SDS-PAGE (Figure 4), the digested samples clearly show peptides at around 3-4 kDa, these are not visible on the IgE-immunoblot. This suggests a low IgE-binding capacity, even though the peptides are of sufficient length to contain one or more IgE-epitopes. As a positive control, intact parvalbumin, both native and glycosylated, binds IgE antibody effectively. Under some blotting conditions, the binding of small peptides to membrane might be hampered, leading to false-negative results. In this study, a PVDF membrane was used, which has been manufactured in a manner to adhere peptides better than predecessors like nitrocellulose. Also, the peptides that are generated by digestion are not sufficiently small that there would be a serious concern of poor binding. Membranes onto which the gels with intact and digested parvalbumin had been blotted were stained for total protein using Ponceau red and had a similar protein profile as the gel (not shown), demonstrating that indeed the peptides were present on the blot. 

The observation that the 3 and 4 kDa peptides do not bind IgE is consistent with the earlier work of Untersmayr et al. who reported that the binding of IgE to codfish extract was virtually absent upon digestion with pepsin for a short time. Although not exactly comparable with our experimental set-up, they also found peptides in the low molecular weight area (estimated at 5 kDa) on electrophoresis that had no IgE-binding [11]. For other food allergens such as Ara h2 from peanut, the peptides remaining after digestion are potent IgE-binders [29], and this may be part of the explanation as to why peanut is a more potent food allergen than fish.

Limited information exists in the literature with regard to the effects of food processing on the allergenicity of fish. One report described that canning reduced the allergenicity of tuna substantially [30]. Another report described that sugar-curing of salmon, a process that may lead to glycosylation and Maillard reactions, results in increased IgE-binding for some sera, while for other sera a decrease is observed [31]. It should be noted that next to parvalbumin, other proteins and potential allergens were present in that experiment. In our hands, the IgE-binding for glycosylated parvalbumin may be slightly decreased based on the somewhat lower intensity observed in Figure 6 (comparing Lanes 1 and 4 for each panel). The lower intensity may also be explained by a more diffuse protein band for glycosylated parvalbumin compared to native, as is also observed on gels stained for protein (Figure 4). In particular for pH 4, the IgE-reactivity of Maillard-treated parvalbumin is lower than expected. This may be due to the solubility behavior of the sample at pH close to the IEP, as suggested above from the far UV CD measurements. Together, the data suggest that the IgE-binding of parvalbumin is not substantially affected by the Maillard reaction. On the other hand, the dimers and higher order multimers present in the Maillard-treated parvalbumin stain intensely on the IgE-immunoblot and are more visible than on the SDS-PAGE (Figures 1(a) and 4). In fact, for native parvalbumin, one can observe some stain for dimers in the IgE-immunoblot experiment (Figure 6), while these bands can hardly be seen in the SDS-PAGE stained for protein (Figure 1(a)). 

The amount of dimers and multimers of parvalbumin in glycosylated material is low as judged by the intensities of the SDS-PAGE (Figures 1(a), 4(b), and 4(c)). However, when stained for IgE-binding, the intensity of the dimers and multimers is as prominent as for the monomeric parvalbumin. This suggests that the dimers and multimers have a stronger IgE-binding than the monomeric parvalbumin. A limitation of western blotting is that comparisons between lanes are qualitative only. Other immunochemical techniques such as ELISA applying IgE could result in quantitative data. Because ELISA cannot distinguish between monomeric and multimeric forms of parvalbumin, this technique is not suitable for attributing IgE-binding to the various forms of our parvalbumin as found in our glycosylated samples. The same is true for immunochemical techniques common in allergy research such as RAST, ImmunoCap, or microarrays. We are therefore limited to the qualitative results of the westernblot. 

The stronger IgE-binding suggests that the dimers (and higher order multimers) are potentially more allergenic than the monomeric form of parvalbumin; however, this needs to be investigated using biological assays, such as basophil histamine-release assays or *in vivo*. It can be speculated that the dimers and multimers of parvalbumin have a more pronounced allergenicity when tested in a biological system, because such systems require the binding of at least two IgE molecules, and this can be facilitated by larger size proteins and multiplication of IgE epitopes. A similar observation was recently made by Vissers et al. [32] who observed that the aggregation of peanut allergen Ara h1 resulted in increased allergenicity as determined in a biological assay (histamine release test), while the IgE-binding to individual epitopes was not increased. 

Our research deals with isolated codfish parvalbumin. It is unknown whether dimers and multimers of parvalbumin will occur as the effect of the Maillard reaction in fish muscle tissue too. Whole tissue extracts are difficult to investigate because the presence of the excess of other (muscle) proteins may interfere in the analytical processes. The western blot applying IgE could be considered because it resolves the monomeric (approximately 12 kDa) and various multimeric forms (approximately 25, 37, 48 kDa). However, the sera from fish-allergic patients commonly also contain IgE to other fish muscle proteins rather than parvalbumin. These proteins have typical molecular weights between 20 and 80 kDa, thereby increasing the complexity of the band pattern on the western blot. Tailor-made analytical methods should be developed to answer the question if glycosylation-induced multimerization of parvalbumin occurs in whole fish tissue too.

## 4. Conclusions

We have prepared different forms of glycosylated cod parvalbumin and characterized them biochemically at different pH mimicking gastric conditions. While some minor differences were observed in the change in secondary and tertiary structures as function of pH between native and glycosylated parvalbumins, the pepsin digestion was comparable for both forms. Primarily peptides of 3 and 4 kDa were formed, and glycosylation had no significant impact on the generation of these peptides. The peptides were no longer able to bind IgE and are therefore considered less allergenic than the intact parvalbumin. In contrast, the glycosylation resulted in small amounts of dimers and higher order multimers with more pronounced IgE-binding. We conclude that food-processing conditions applied to fish allergen can potentially lead to an increase in allergenicity, even while the protein's digestibility is not affected by such processing. Therefore, the allergenicity of fish products should be monitored with great care when food-processing steps are used that may induce glycosylation. 

## Figures and Tables

**Table 1:** Zero-crossings (in nm) observed in far UV-CD spectra of native and glycosylated parvalbumins at different pHs.

	pH 1.2	pH 2.0	pH 3.0	pH 4.0	Neutral pH
Native parvalbumin	<*	198.6	203.5	206.7	204.2
5-hour glycosylated parvalbumin	201.9	201.8	203.5	204.4	203.8
12-hour glycosylated parvalbumin	202.5	202.3	203.7	204.4	203.2

*A reliable value could not be determined because the high chloride concentration at this pH did not allow to record below 200 nm. At 200 nm, the ellipicity was still negative.

## Abbreviations

AGEs: Advanced glycosylated end products
IEP: Isoelectric point
MALDI-TOF MS: Matrix-assisted laser desorption ionizationtime of flight mass spectroscopy
OPA: Orthophthaldialdehyde.
